# Stem cells differentiation into insulin-producing cells (IPCs): recent advances and current challenges

**DOI:** 10.1186/s13287-022-02977-y

**Published:** 2022-07-15

**Authors:** Isaura Beatriz Borges Silva, Camila Harumi Kimura, Vitor Prado Colantoni, Mari Cleide Sogayar

**Affiliations:** 1 Cell and Molecular Therapy Center (NUCEL), School of Medicine, University of São Paulo, São Paulo, SP 05360-130 Brazil; 2 Department of Biochemistry, Chemistry Institute, University of São Paulo, São Paulo, SP 05508-000 Brazil

**Keywords:** Insulin-producing cells (IPCs), Type 1 diabetes mellitus (T1D), Stem cells differentiation, Cell therapy

## Abstract

Type 1 diabetes mellitus (T1D) is a chronic disease characterized by an autoimmune destruction of insulin-producing *β*-pancreatic cells. Although many advances have been achieved in T1D treatment, current therapy strategies are often unable to maintain perfect control of glycemic levels. Several studies are searching for new and improved methodologies for expansion of *β*-cell cultures in vitro to increase the supply of these cells for pancreatic islets replacement therapy. A promising approach consists of differentiation of stem cells into insulin-producing cells (IPCs) in sufficient number and functional status to be transplanted. Differentiation protocols have been designed using consecutive cytokines or signaling modulator treatments, at specific dosages, to activate or inhibit the main signaling pathways that control the differentiation of induced pluripotent stem cells (iPSCs) into pancreatic *β*-cells. Here, we provide an overview of the current approaches and achievements in obtaining stem cell-derived *β*-cells and the numerous challenges, which still need to be overcome to achieve this goal. Clinical translation of stem cells-derived *β*-cells for efficient maintenance of long-term euglycemia remains a major issue. Therefore, research efforts have been directed to the final steps of in vitro differentiation, aiming at production of functional and mature *β*-cells and integration of interdisciplinary fields to generate efficient cell therapy strategies capable of reversing the clinical outcome of T1D.

## Introduction

### Diabetes mellitus

Diabetes mellitus (DM) is a metabolic disease, which arises from a complete deficiency of insulin production—type 1 diabetes (T1D)—or inability to utilize this hormone, as occurs in type 2 diabetes (T2D). It is among the top 10 causes of death in adults, being estimated to have caused 4.2 million deaths globally in 2019 [1]. According to the International Diabetes Federation (IDF) [1], approximately 463 million adults aged 20–79 years old are living with diabetes. An additional number of 1.1 million children and adolescents under 20 years old live with T1D. The IDF [1] also estimates that, by 2045, 700 million adults will be living with DM globally. Therefore, it is crucial to discover and understand the underlying mechanisms of this disease, as well as searching for new and more efficient alternative therapy strategies.

T1D is characterized by the autoimmune destruction of insulin-producing *β*-pancreatic cells. Autoreactive T cells are key mediators of *β*-cell destruction, resulting in a complete depletion of insulin hormone, which is essential for carbohydrate metabolism and regulation of normal blood sugar (glycemic) levels [2]. The balance between activated autoreactive memory/effector T cells (Teffs) and activated regulatory T cells (Tregs) is critical for maintaining a healthy immune status. The mechanisms of autoimmunity in T1D are driven by activation of the Teffs, leading to initiation or exacerbation of a preexisting autoimmune process. The persistent activation of Teffs, uncontrolled by Tregs, leads to chronic inflammation and immune response to *β*-pancreatic cells [2, 3].

According to the World Health Organization (WHO) [4], T1D is responsible for approximately 10% of the totality of DM cases in the World. In contrast, T2D is characterized by the development of insulin resistance due to alterations in the cell insulin receptor or in intermediate mediators of the insulin signaling pathway. The prolonged dysregulation of glycemic levels can cause several chronic health complications, such as diabetic nephropathy, cardiovascular diseases (heart attack, stroke and peripheral artery disease), retinopathy and diabetic neuropathy, which can lead to amputation of inferior members and even death.

### T1D therapies

Despite all efforts placed on T1D research throughout the years, the cure for this disease still remains more of an aspiration. Insulin therapy constitutes the main form of treatment for T1D patients; however, continuous administration of exogenous insulin requires an intensive therapeutic regimen and frequent monitoring of glycemic levels, with limited degrees of effectiveness. In addition, this regimen does not accurately mimic the endogenous insulin secretion kinetics; therefore, it is not able to efficiently prevent some of the deleterious effects of hyperglycemia. Moreover, even though insulin therapy slows down the development of secondary complications, it is not able to control glycemic levels in hyper-labile patients [5], who are subject to a wide variation in glycemic rates, showing severe and often fatal hypoglycemic episodes, even under the best conditions of glycemic monitoring and insulin administration [5].

Some advances were made in the field of insulin administration, with the creation of alternative administration routes, such as inhalable insulin preparations, which have become clinically feasible [6, 7], and in the field of glucose level monitoring, with the creation of devices that utilize capillary blood samples [8]. However, it is still necessary to search for other alternative therapeutic strategies to improve the patient’s quality of life and enable a less strict and stressful regimen. From a physiological point of view, restoration of *β*-pancreatic cell functions through transplantation of insulin-producing tissue (whole pancreas or isolated pancreatic islets) may be the best therapeutic option so far.

### Therapeutic alternatives for T1D

According to Fioretto et al*.* [9], whole organ pancreas transplantation is a viable therapeutic option, since it improves the patient’s quality of life and promotes regression of some late complications associated with T1D. However, this procedure constitutes a major surgical intervention, which requires a strict immunosuppressive regimen and heavily depends on properly functioning of the donor pancreas for a successful treatment, being recommended only for patients with brittle/labile T1D who also need a kidney transplant [10]. Pancreatic islets transplantation, introduced in Brazil by our research group [11, 12], has been shown to be a promising alternative to whole organ pancreas transplantation, since it is a simpler and less invasive procedure. According to Hering et al*.* [13], transplantation of pancreatic islets is a safe and efficient treatment option for T1D patients with hypoglycemia. Nevertheless, there are still some factors that limit this procedure, such as the low availability of pancreas donors and the requirement for constant patient immunosuppression [10, 14].

Chronic usage of immunosuppressant medication becomes necessary for immunological acceptance of the islet allograft; however, this regimen is associated with various side effects, such as oral sores, gastrointestinal diseases, hypertension, dyslipidemia, anemia, increased infection susceptibility, cancer and systemic toxicity [15]. Therefore, encapsulation of pancreatic islets has emerged as a promising strategy to avoid the need for these immunosuppressive drugs. Production of semipermeable microcapsules for biological application, containing cells or proteins, was initially suggested in the 90’s [16], but considerable progress has been achieved in the field since then, with a major increase in application possibilities, including as an alternative for T1D treatment.

To avoid using steroid-based agents that damage *β*-cells and are known to be diabetogenic or induce peripheral insulin resistance, a glucocorticoid-free immunosuppressive protocol was developed by the Shapiro’s Group [17], for usage in islet transplantation trials. This protocol includes sirolimus, low dosage of tacrolimus and a monoclonal antibody against the interleukin-2 receptor (daclizumab). Their findings, in a study with T1D patients, indicate that islet transplantation alone is associated with minimal risks for the patient and results in good metabolic control, with normalization of glycated hemoglobin values and restricted requirement for exogenous insulin [17]. This protocol, known as the Edmonton Protocol, was considered as a breakthrough, becoming the standard procedure for islet transplantation, constituting a promising step toward the development of a cure for T1D [18]. However, the standard procedure for pancreatic islets transplantation is based on isolation and purification of islet cells from deceased donors, a process that requires two to four donors per patient, since the efficiency of islet isolation is well below 100% and, additionally, only about 50% of the implanted islets survive after transplantation [19]. In addition, several factors interfere with the viability of the graft after transplantation, such as quality of the donated organ, viability and functionality of the purified islets and the patient’s own immune response [20]. Although many advances have been reached in the field, the need for a large number of viable islets, along with the low availability of donors, is still an important factor that compromise the viability of this methodology.

Although progress has been made, pancreas and islet transplantation are still limited by the limited number of pancreas donors, chronic immunosuppression, which causes a number of adverse effects, and, also, by the recurrence of autoimmunity/onset of alloimmunity [21]. Therefore, a variety of T1D immunotherapy approaches have been developed aiming to prevent or delay T1D onset in predisposed individuals or preserve insulin production in T1D patients [22–24]. A hallmark of T1D is the emergence of *β*-cells destructive autoantibodies against endogenous antigens, which include proinsulin (biosynthetic precursor of insulin), proinsulin C-A junction (connection of C-peptide and A chain of proinsulin), glutamic acid decarboxylase 65 (GAD65, tolerogenic vaccine for T1DM prevention), islet antigen 2 (IA-2) and zinc transporter 8 (ZnT8) [25–29]. Therefore, the overarching goal of immune-focused therapies in T1D is to prevent or delay the loss of functional *β*-cell mass.

Immunotherapies directed to T1D can be classified into non-autoantigen-specific and autoantigen-specific interventions [30]. Non-antigen-specific treatments are based upon the premise that enhancing immune regulatory mechanisms can ameliorate the destructive autoreactive immune responses, including those against *β*-cells. A large clinical trial was carried out investigating the therapeutic utility of cyclosporin A in the late 80s. Although cyclosporin A treatment increased T1D remission, this was only for a short duration, since the studies reported progressive increase in daily insulin requirement [31]. Similarly, there have been many clinical interventional studies carried out using anti-CD3 and anti-CD2034 monoclonal antibodies [32]. However, only transient preservation in C-peptide levels was observed [33]. Furthermore, a study investigating safety and efficacy of anti-thymocyte globulin (ATG) failed to preserve *β*-cell function after two years [34].

Compared to non-autoantigen-specific immunomodulation, autoantigen-specific immunotherapy is expected to selectively modulate T1D-related autoimmunity while preserving the global immune homeostasis intact [35–37]. There are studies related to modulation of autoantigen-specific T cell, such as Santamaria et al., 2016 that developed nanoparticles coated with autoantigen-related MHC-II/peptide complex molecules (pMHCII) [36]. There are also trials related to autoantigen-specific B lymphocyte modulation, which have been shown to be more promising than non-specific inhibition of B lymphocytes, for example, by depleting insulin-reactive B cells [37]. Significant progress has already been made through either non-autoantigen-specific immune modulation or T1DM autoantigen-specific immunotherapy. Nevertheless, so far, no T1DM immunotherapy is yet available to replace the standard insulin replacement therapy [30].

Another possible alternative for T1D cell therapy is based on using human mesenchymal stem cells (MSCs) due to their ability to release immunomodulatory molecules that may interrupt the early *β*-cell destruction by the patient’s own immune system [38]. This may be achieved by infusion of MSCs, which may be obtained from various tissues, directly into the patient’s bloodstream or by apheresis, followed by ex vivo stem cell Educator Therapy, in which the patient's blood passes through a closed-loop system that separates white blood cells, which are momentarily co-cultured with stem cells, before returning them to the patient's bloodstream [39].

Transdifferentiation has also become a potential method to produce functional *β*-cells. Some findings indicate that, under certain conditions, pancreatic cells, such as acinar and ductal cells, can transdifferentiate into *β*-cells, following viral transduction [40] or in response to soluble factors [41–43]. Nevertheless, further research is required to understand this transdifferentiation of non-*β* cells into insulin-producing cells (IPCs), since it remains unclear how similar reprogrammed cells are with respect to endogenous *β*-cells [44].

Several studies have been directed at new and improved methodologies for expansion of *β*-cell cultures in vitro*,* aiming at increasing the supply of IPCs for pancreatic islets replacement therapy. Since the nature of T1D disease is a dysfunction of only one cell type, *β*-cell, differentiation of pluripotent stem cells in *β*-like cells or IPCs represents a promising approach for T1D cell replacement therapy [45]. Stem cells display two main characteristics: They are non-specialized cells that self-renew for long periods of time, through the cell division cycle, without differentiating into other cell types, while maintaining their capacity to differentiate into different cell types, according to the physiological and experimental conditions to which they are submitted [46].

Achieving economically and technologically viable stem cell-derived therapies still constitutes a great challenge, which requires strict rules for handling and production under appropriate current Good Manufacturing Practice (cGMP) conditions. A cGMP facility is a production facility that includes the manufacturing space, the storage warehouse for raw and finished product and support laboratory areas, also including quality control and quality assurance programs, establishing a Quality System approach [47]. Implementation of procedures and protocols adapted to cGMP requirements is critical to ensure robust and consistent high-quality stem cell manufacturing.

**Fig. 1:**
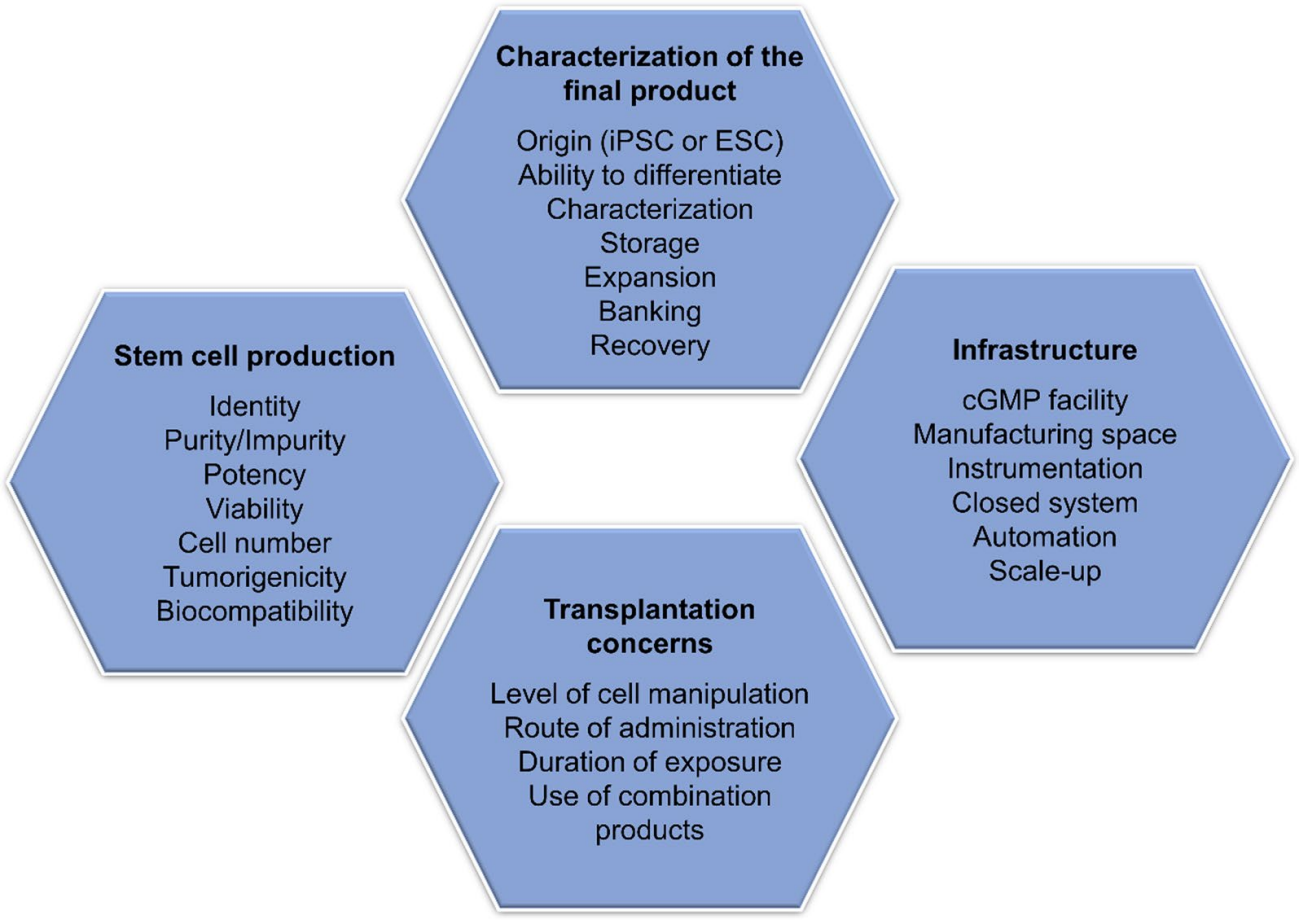
Overview of the relevant requirements for institution of stem cell-derived therapy clinically, including *β*-like cells. cGMP: Current good manufacturing practice. iPSC: Induced pluripotent stem cell; ESC: embryonic stem cell

To ensure uniformity from batch-to-batch, manufacturers are required to keep Master Batch Records (MBRs) and Batch Production Records (BPRs) [48]. Detailed standard operating procedures (SOP) and MBRs for manufacturing of stem cell-derived *β*-cells lots suitable for clinical transplantation are key to ensure that a viable mass of insulin-producing cells can be safely infused into the recipients. These SOPs detail each procedural step, from stem cell expansion and differentiation in vitro to pre-transplantation, quality controls and product release criteria for transplantation [49] ensuring that the reproducibility of the final product is in accordance with established specifications [50]. Also, operation in a closed system and automation of the manual steps enable sterility, processing robustness and reproducibility [48]. The main requirements for stem cell clinical-grade manufacturing, product characterization, infrastructure and concerns related to therapeutic application are shown in Fig. 1. Importantly, investigational products must go through a thorough review process by a regulatory agency, such as FDA (Food and Drug Administration), EMA (European Medicines Agency) and ANVISA (Brazilian Health Regulatory Agency), to determine the safety and effectiveness of products in a well-controlled clinical trial with human subjects.

Based on ongoing clinical trials using stem cell-derived *β*-cells, the eligibility criteria for participating in a clinical study usually include age 18–65 years, clinical history of T1D with > 5 years of duration, episodes of severe hypoglycemia and stable diabetes treatment [51]. During the clinical study, a protocol for outcomes measurement is established in order to evaluate the effectiveness of the transplantation, with most clinical studies having one primary outcome measure, but some have more than one. Graft function depends on the complex physiologic relationship between the graft and the recipient, with several metabolic tests being necessary to monitor graft function and the success of the transplantation. Similar to islet transplantation, the primary endpoints for stem cell-derived *β*-cells should consist of normal HbA1c level (HbA1c ≤ 6.5%), absence of hypoglycemic episodes and graft durability. The major secondary endpoints include insulin independence, stimulatory test using meal tolerance test (MTT) and oral glucose tolerance test (OGT), continuous glucose monitoring and patient quality of life [52, 53].

Many of the previously mentioned therapeutic strategies, exemplified in Table 1, are currently being clinically tested. Strategies focused on immunomodulation by MSCs infusion are the most highly represented among cell therapies for T1D currently in clinical trials. The combination of immunomodulatory and regenerative properties of MSCs made these cells the most frequently investigated stem cells for clinical applications during the last couple of decades [54]. The immunoregulatory mechanism mediated by MSCs is based on inhibition of effector T cells and other immune cells, while inducing Tregs, reducing directly and indirectly the production of pro-inflammatory cytokines. Many immunosuppressive cells, such as Tregs, regulatory B cells (Bregs), endothelial progenitor cells (EPCs) and myeloid-derived suppressor cells (MDSCs), express TNFR2, TNFα receptor, in direct relationship to their immunosuppression efficiency [55]. In fact, Beldi et al. [56] showed that mouse TNFR2 KO-MSCs have significantly lower immunosuppressive and immunomodulatory effect against T cells. It was further demonstrated that TNFR2 blockade led to increased levels of IFN*γ*, TNF*α* and IL-6 pro-inflammatory and decreased IL-10 and TGF*β* anti-inflammatory cytokines and nitric oxide production. Moreover, TNFR2 deficiency leads to the induction of Tregs with remarkably less immunosuppressive effect [54]. It has also been suggested that mast cells could confer resistance to T1D, by promoting increased Treg cells, and decreased IL-17-producing T cells in the pancreatic lymph nodes [57]. Considering the autoimmune nature of T1D, marked with a disbalance in Teff and Tregs, as previously described, the MSCs and other molecules that boost Tregs responses represent a therapeutic option for immunomodulation to improve T1D outcomes.

**Table 1:** Main therapeutic strategies for T1D in current clinical trials

Therapeutic strategy	Sponsor	Trial status	Phase	Country	Identifier	Outcome
Allogeneic Wharton´s jelly derived mesenchymal stromal cells transplantation	NextCell Pharma	Completed	Phase I/II	Sweden	NCT03406585 (Clinical Trials)	Preserved *β*-cell function in newly diagnosed T1D patients
Allogeneic umbilical cord-derived mesenchymal stromal cells (UC-MSCs) transplantation	Medical University of South Carolina	Ongoing	Phase I	USA	NCT04061746 (Clinical Trials)	–
Allogeneic adipose-derived mesenchymal stromal cells and autologous bone marrow mononuclear cells co-transplantation	Sophia Al-Adwan (University of Jordan)	Ongoing	Phase I	Jordan	NCT02940418 (Clinical Trials)	–
Ex vivo cultured adult allogeneic MSCs (PROCHYMAL) transplantation	Mesoblast Inc	Completed	Phase II	USA	NCT00690066 (Clinical Trials)	No results posted
Autologous bone marrow-derived mesenchymal stromal cells transplantation	Royan Institute	Ongoing	Phase I/II	Iran	NCT04078308 (Clinical Trials)	–
Dental Pulp MSCs from human exfoliated teeth transplantation	Shanghai CAR-T Biotechnology Co., Ltd	Ongoing	Phase I	China	NCT03912480 (Clinical Trials)	–
Autologous hematopoietic stem cells transplantation	Dr. Olga Graciela Cantu Rodriguez (Dr. Jose E. Gonzalez University Hospital)	Completed	Phase I/II	Mexico	NCT01121029 (Clinical Trials)	No results posted
Autologous hematopoietic stem cells transplantation with immunosuppression regimen	The Affiliated Nanjing Drum Tower Hospital of Nanjing University Medical School	Completed	Phase II	China	NCT01341899 (Clinical Trials)	Modulated lymphocytes and preserved *β*-cell function in Chinese patients with newly onset of type 1 diabetes and diabetic ketoacidosis
Bone marrow-derived hematopoietic stem cells transplantation with immunosuppression regimen	University of São Paulo Ribeirão Preto Medical School	Completed	Phase I/II	Brazil	NCT00315133 (Clinical Trials)	No results posted
Reversal of Type 1 Diabetes in Children by Stem Cell Educator Therapy	Tianhe Stem Cell Biotechnologies Inc	Ongoing	Phase I/II	China	NCT01996228 (Clinical Trials)	–
Autologous expanded progeny of an adult CD34 + stem cell subset (InsulinCytes) transplantation	Imperial College of London	Completed	Phase I	UK	NCT00788827 (Clinical Trials)	No difference was observed in patients' conditions after treatment
Implantable device with insulin-producing cells transplantation under immunosuppression regimen	Sernova Co	Ongoing	Phase I/II	USA	NCT03513939 (Clinical Trials)	–
Human allogeneic pancreatic islet cells transplantation with steroid-free immunosuppression regimen (Edmonton Protocol)	Emory University	Completed	Phase II	USA	NCT00133809 (Clinical Trials)	62.5% insulin-independent subjects one year after intervention 50% subjects with HbA1C ≤ 6.5% after 5 years and 37,5% subjects exhibiting fasting C-peptide levels ≥ 0.5 ng/mL after 5 years
Pancreatic islet cells endoscopic transplantation into the gastrointestinal sub-mucosa with immunosuppressive regimen	University of California (San Francisco)	Ongoing	Phase I	USA	NCT02402439 (Clinical Trials)	–
Human allogeneic pancreatic islet cells transplantation under immunosuppression regimen	National Institute of Allergy and Infectious Diseases (NIAID)	Completed	Phase III	USA	NCT00434811 (Clinical Trials)	No results posted
Sequential transplantation of umbilical cord-derived blood stem cells and pancreatic islet cells	Children’s Hospital of Fudan University	Ongoing	NA	China	NCT03835312 (Clinical Trials)	–
Allogeneic islet cells transplantation into the omental pouch	University of Alberta	Completed	Phase I/II	Canada	NCT02821026 (Clinical Trials)	No results posted
Allogeneic pancreatic islet cells and parathyroid gland co-transplantation	Peter Stock (University of California, San Francisco)	Ongoing	Phase I/II	USA	NCT03977662 (Clinical Trials)	–
Encapsulated stem cell-derived pancreatic islet cells transplantation	ViaCyte	Completed	Phase I	Canada	NCT03162926 (Clinical Trials)	No results posted
A Safety, Tolerability and Efficacy Study of VX-880	Vertex Pharmaceuticals	Ongoing	Phase I/II	USA	NCT04786262 (Clinical Trials)	–

Only two trials involve the usage of pluripotent stem cells fully or not differentiated into insulin-producing *β*-cells, which includes two companies, namely ViaCyte and Vertex. The ViaCyte initiative is considered as the first cell replacement therapy in clinical trials, with islet-like cells derived from stem cells, testing the safety and efficacy of pancreatic precursor cells incorporated into its encapsulation devices, namely PEC-Encap and PEC-direct. The biologically active component of the PEC-Encap and PEC-direct product candidate is stem cell-derived pancreatic islet cell progenitors, called PEC-01^™^ cells. ViaCyte has shown that once implanted and engrafted, the cells mature into *β* cells and other islet cell types and are able to secrete insulin in a regulated manner. The PEC-Encap was developed with the purpose of eliminating the need for immunosuppression. The device was evaluated in a 24-month open-label, dose-escalating Phase 1/2 study in T1D patients with minimal insulin-producing *β*-cell function. The potential for prolonged cell survival has been demonstrated, for as long as 24 months, but has been inconsistent among subjects and primarily limited by a foreign body response to the device component which indicates the requirement for optimization of the device materials [58]. PEC-Direct is an islet cell replacement therapy comprised of stem cell-derived pancreatic islet progenitor cells in a pouch that allows direct vascularization of the implanted cells, thus requiring a concomitant immunosuppressant regimen. A report analysis of data from the first cohort of 15 patients showed that up to one year, patients had 20% reduced insulin requirements, spent 13% more time in target blood glucose range, had stable average HbA1c < 7.0% and had improved hypoglycemic awareness. Implantation of PEC-01 cells was well tolerated, and the serious adverse events that impacted two patients have been previously documented to be associated with the immunosuppression protocol. Only one patient had a > 50% reduction in insulin requirements within one year post-implantation, and no patients achieved insulin independence [59].

Recently, a report by the Vertex company announced positive day 90 data for the first patient from the Phase 1/2 clinical trial of VX-880, an investigational stem cell-derived, fully differentiated pancreatic islet-like cell replacement therapy. This patient had a 91% decrease in daily insulin requirement and simultaneous robust improvements in glucose control, indicating that treatment was generally well tolerated. This was the first demonstration of patient with T1D achieving robust restoration of insulin production from such a cell therapy. The patient was treated with a single infusion of VX-880 at half the target dose in conjunction with immunosuppressive therapy. There were no serious adverse events related to VX-880, and the majority of the adverse events were considered mild to moderate1 [60].

The other trials depicted in Table 1 involve pancreatic islet cell transplantation, based on the Edmonton Protocol or variation thereof, in combination with an immunosuppression regimen (NCT00133809; NCT00434811) or evaluation of different transplantation sites (NCT02402439; NCT02821026) or the combination with other non-endocrine tissues (NCT03977662). Considering the already available advances in the pluripotent stem cells area and the advantages that stem cells-derived IPCs could provide for T1D treatment, these data highlight the crucial necessity to establish efficient and reproducible protocols for stem cell differentiation into IPCs in order to enable their clinical applicability. Therefore, the aim of this review is to provide an overview of the current approaches and achievements in obtaining stem cells-derived IPCs in vitro and the challenges which still need to be overcome.

## Stem cells as a source of insulin-producing cells

### Stem cells

Stem cells (SCs) are non-specialized cells capable of both auto-renewal and differentiation into different cell types [61]. The cell differentiation process depends on the physiological or experimental conditions to which the cells are subjected, being induced, on the one hand, by intracellular factors, such as expression of key genes, and, on the other, by extracellular factors, such as differentiation-inducing molecules present in the cellular microenvironment [62].

SCs can be classified into three main types: embryonic SCs, adult SCs and induced pluripotent SCs. Embryonic stem cells (ESCs) comprise a class of stem cells derived from the inner cell mass of the blastocyst. ESCs are pluripotent cells that can generate cells from all three embryonic leaflets (endoderm, mesoderm and ectoderm); therefore, they have the greatest potential for cell differentiation [63, 64]. Adult stem cells (ASCs) are non-differentiated cells found in most specialized adult tissues, being able to generate only a selection of cell types of those which are present in that tissue, mainly due to their maintenance and self-renewal [41]. Although displaying a lower proliferation and differentiation potential, ASCs present the great advantage of enabling autologous transplantation [65, 66].

Induced pluripotent stem cells (iPSCs) are genetically modified and reprogrammed cells that originate from adult cells through cellular genetic modification mechanisms, generating cell products, which are similar to ESCs [67]. The reprogramming process is based on transfection of transcriptional factor genes (Oct4, Sox2, c-Myc and Klf4), which are highly expressed in ESCs, through retroviral transduction [68]. After introduction of these reprogramming factors, it is possible to obtain, from differentiated adult cells, groups of cells that are similar to human ESCs, regarding their morphology, cell proliferation rate, antigenic profile, gene expression profile, epigenetic profile, telomere activity and differentiation capacity.

## In vitro stem cell differentiation into IPCs as a therapeutic strategy for T1D

In vertebrates, the embryonic pancreas originates from dorsal and ventral protrusions which branch out of the primitive gut. The two pancreatic buds then grow and merge to form the definitive pancreas [69]. The adult pancreas is a retroperitoneal gland divided into three parts: the head (proximal), body and tail (distal). The pancreatic gland has two main cellular compartments with distinct functions, namely the exocrine and the endocrine compartments. The exocrine pancreas, mainly constituted by acinar cells, is responsible for the production and secretion of digestive enzymes, such as proteases, lipases and nucleases, and corresponds to most of the pancreatic mass [70, 71]. In contrast, the endocrine pancreas represents only a small percentage (1–2%) of the entire organ, with cells being organized into cellular groups called islets of Langerhans, which are embedded into the exocrine tissue. The endocrine pancreas consists mainly of four cell types, namely *⍺*, *β*, *δ* and PP cells, which produce, respectively, the glucagon hormone, insulin hormone, somatostatin hormone and the pancreatic polypeptide [69, 70].

iPSCs and ESCs are ideal candidates for differentiation into *β*-cells due to their outstanding renewal ability, which enables the generation of high numbers of cells that have long been sought in the clinic [71]. In general, the main objectives to be achieved during the differentiation process are: (a) identification of stem cells or progenitor lineages that are capable of self-renewal and differentiation; (b) identification of proliferative signals as well as instructive signals that induce the differentiation process; and (c) identification of molecular signals that maintain the correct physiological state and viability of the differentiated cells [69].

Different strategies have been adopted to obtain IPCs, namely spontaneous differentiation with further selection of Nestin + progenitor cells [72], inhibition of phosphatidylinositol-3-kinase (PI3K) [73], mimicking the in vivo developmental process by adding differentiation factors [74–77], co-culture with fetal pancreatic buds or culture in the presence of fetal pancreas conditioned medium [78] or transgenic expression of pancreas-specific transcription factors, such as *foxa2, ptf1a, pdx1, hnf4a* (hepatocyte nuclear factor 4 alpha), *hnf6* (hepatocyte nuclear factor 6), *ngn3, pax4, neuroD1* and *nkx6.1* [71, 79]. Currently, differentiation protocols have been designed using consecutive cytokines or signaling modulators treatments, in specific doses, to activate or inhibit the main signaling pathways that control the differentiation of iPSCs into pancreatic *β*-cells, namely Wnt; Nodal/Activin A; BMPs; FGF; EGF (epidermal growth factor); Hedgehog; retinoid; and Notch (Fig. 1) [80]. Obtaining mature IPCs in vitro depends on a refined control of concentration, time and duration of treatment with the defined growth and differentiation factors.

### Embryoid bodies (EBs)

**Fig. 2:**
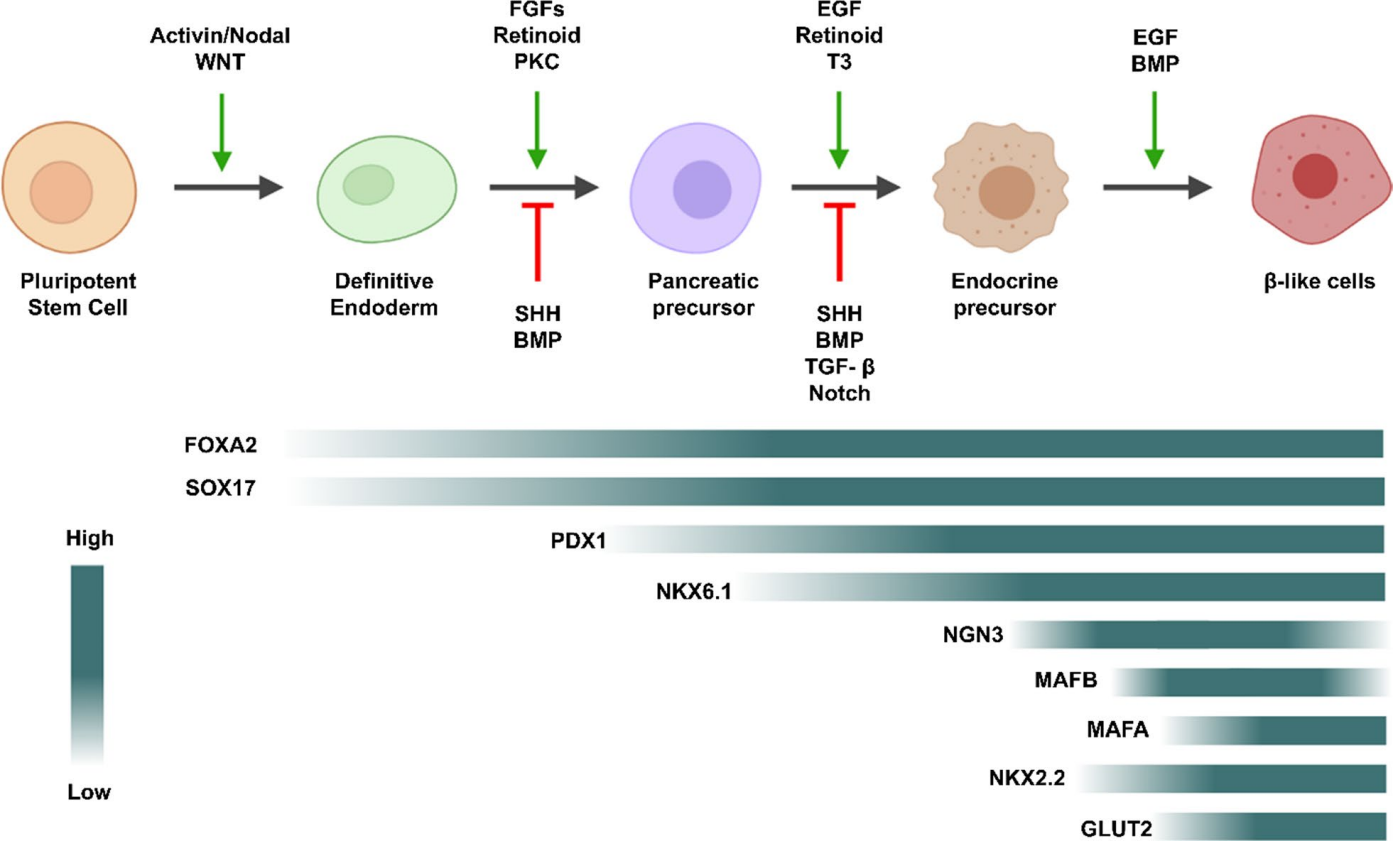
Schematic representation of the signaling pathways that coordinate each step of *β*-cell differentiation and expression levels of the main transcription factor and functional proteins during *β*-cell differentiation and maturation. NGN3 and MAFB are transiently expressed, while the others remain expressed after maturation. BMP: Bone morphogenetic protein; EGF: Epidermal growth factor; FGF: Fibroblast growth factor; PKC: Protein Kinase C; SHH: Sonic hedgehog; T3: Triiodothyronine; and TGF-*β*: Transforming growth factor beta

One of the first steps of the differentiation protocol is the formation of embryoid bodies (EBs), which is necessary to mimic the in vivo embryonic stage of cellular organization. The EBs spontaneously differentiate into cell types of all three primary germ layers, namely ectoderm, mesoderm and endoderm. The EBs formation stage is described as being crucial for determination of the final cells differentiation potential to generate IPCs. Depending on the size of the EB, there is a greater probability of obtaining precursor cells of different cell types [81, 82]. Because the number of specifically differentiated cell types is relatively low after spontaneous differentiation, the following steps aim to induce different signaling pathways to promote cell differentiation and specification. On this basis, the subsequent stages are the formation of definitive endoderm, followed by pancreatic progenitors, pancreatic endocrine cells and, finally, *β*-cells. Differential gene expression analysis during this process should be useful to follow the in vitro differentiation stages (Table 2; Fig. 2).

**Table 2:** Function of the main genes involved in *β*-cell differentiation

Gene	Function	References
*nanog (Nanog homeobox)*	Critical for early embryogenesis and for ESC pluripotency	[83, 84]
*oct4 (POU domain, class 5, transcription factor 1*		
*sox2 (SRY (sex determining region Y)-box 2*		
*foxa2 (forkhead box A2)*	Necessary for proper endoderm formation and *pdx1* expression. Also required for regulated insulin secretion in mature *β*-cells—regulates the expression of important genes for glucose sensing in pancreatic *β*-cells and glucose homeostasis	[85–89]
*cxcr4 (chemokine (C-X-C motif) receptor 4)*	Required for proper *β* cells generation – it is a key marker of definitive endoderm, controlling cells migration during gastrulation	[90, 91]
*sox17 (SRY (sex determining region Y)-box 17*	Controls segregation of liver, biliary system, and pancreas; regulates insulin trafficking and secretion in *β*-cells	[92–95]
*nkx6.1 (NK6 homeobox 1)*	Directly targeted genes involved in insulin biosynthesis (Slc30a8 and Ero1lb), glucose transporter 2 (Glut2), and glucose metabolism	[96]
*pdx1 (pancreatic and duodenal homeobox 1*	Mainly involved in glucose-dependent regulation of insulin gene expression. Also necessary for the activation of several genes, including insulin, somatostatin, glucokinase, islet amyloid polypeptide and GLUT2	[97, 98]
*ptf1a (pancreas associated transcription factor 1a*	Required for exocrine cell formation—activates an acinar cell genes repertoire. Has a complex set of interactions with Notch downstream intercellular mediators to regulate target patterning genes and acinar-specific genes	[99–102]
*sox9 (SRY-box transcription factor 9)*	Necessary for regulation of pancreatic specification, differentiation and duct morphology	[103–107]
Cg (Chromogranin) A	Constitute the regulated pathway of protein hormone secretion including all four pancreatic peptide hormones and gastrin. It is involved in the generation of secretory granules and is considered a pan-endocrine marker	[108, 109]
*ngn3* *(neurogenin 3)*	Endocrine formation key regulator—induces the expression of endocrine genes such as *neuroD1*, *nkx2.2*, *nkx6.1*, *pax4*, *pax6* and *isl1*	[110–112]
*neuroD1* *(neurogenic differentiation 1)*	Involved in islet growth, proliferation and endocrine differentiation in pancreatic progenitors. Activates IA1 (Insulin Associated 1), a zinc finger protein that appears to be important in executing the endocrine differentiation process. Can activate the *pax6* gene	[113–116]
*nkx2.2* *(NK2 homeobox 2)*	Necessary for *β*-cell precursors to express *nkx6.1* and *ins*. Also binds to and activates *mafa*	[117–119]
*pax4* *(paired box gene 4)*	Directs formation of *β* and δ cells. Acts as a transcriptional repressor, being especially effective for ghrelin expression and pax6-mediated glucagon expression	[120–124]
*ins (insulin)*	Provides instructions for producing the insulin hormone	[125]
*mafa (v-maf musculoaponeurotic fibrosarcoma oncogene family, protein A)*	Controls and activates insulin gene expression	[126, 127]
*mafb (v-maf musculoaponeurotic fibrosarcoma oncogene family, protein B)*	Appears to be a key regulator of *α*- and *β*-cell maturation, since Mafb binds to and activates the *mafa* gene, causing a transition from *mafb* to *mafa* expression in insulin + cells as they transition from immature to mature *β* cells	[128]
*Glut2 / SLC2A2* *(solute carrier family 2 member 2)*	An integral plasma membrane glycoprotein of islet *β*-cells that mediates facilitated bidirectional glucose transport	[129]

### Definitive endoderm (DE)

Initially, factors that lead to activation of the Nodal pathway are employed, since the signaling gradient of these factors leads to endoderm (high nodal) and mesoderm (low nodal) segregation, thus displaying a key function in endodermal formation [130]. Activin A, a member of the TGF-*β* superfamily (transforming growth factor *β*), is described as a crucial activation factor for the Nodal pathway [129]. Nodal-mediated signaling modulates the FGF, BMP and Wnt pathways, activating the gastrulation process [131]. Therefore, activin A may be used to mimic Nodal activity in vitro. Expression of Sonic hedgehog (SHH), a potent intercellular patterning signal, is strikingly absent from pancreatic endoderm. Hebrook et al. [132] showed that activin signaling, as a notochord factor, can decrease Shh expression, while inducing expression of Pdx1 and insulin by chick endoderm, thereby permitting pancreas development. However, some studies have shown that activin A may also induce neuronal cells [133]. Therefore, one of the most important parameters for efficient endoderm differentiation is definition of the activin A concentration [129]. Retinoic acid also plays a crucial role in endoderm development during a step between endoderm formation and pancreatic progenitors’ specification [134].

Once formed, definitive endoderm generates the gut tube, which is patterned into anterior and posterior fates by gradients of WNT, FGF and retinoic acid (RA) signaling [135]. WNT signaling is described to have a direct and multifaceted role for WNT signaling in intestinal specification and patterning. WNT signaling acts directly on definitive endoderm to induce Cdx2, a major regulator of intestine-specific genes involved in cell growth and differentiation [136]. Reports demonstrated the ability of WNT to cooperate with Activin signaling to promote definitive endoderm formation, where the optimal induction of differentiation in definitive endoderm was achieved in cells simultaneously treated with Wnt3a [74, 137–140]. However, Kunisada et al. [141] found that treatment with activin A plus CHIR99021 induced SOX17 and FOXA2 double-positive definitive endoderm more efficiently, when compared with activin A plus Wnt3a.

### Pancreatic progenitors

The next step is to induce pancreatic precursor cells, which are cells that display the potential to give rise to all pancreatic lineages and originate all the functional endocrine and exocrine cell types. Considering that the mesenchymal tissues have a critical importance for growth of all pancreatic cell lineages, studies indicate that the FGF signaling pathway, derived from the surrounding mesenchymal tissue, is essential for the formation of specific cell domains. FGF10, as a mesenchymal factor, has an indispensable role in development of the pancreatic epithelium, acting as a mitogenic factor to stimulate proliferation and allowing amplification of pancreatic cells in vitro [142, 143]. It has been demonstrated that culture of dissociated endodermal cells at lower density, followed by longer retinoic acid and FGF10 signaling, results in a high yield of pancreatic progenitors expressing key markers, such as Pdx1 and Nkx6.1 [144]. Ostrom [145] also provides support for an intrinsic role for retinoic acid signaling in specified Ipf1/Pdx1 + pancreatic progenitor cells. FGF2 or basic FGF, known as a notochordal signal, can affect this phase, since it maintains pdx1 expression in the endoderm and potentiates *β*-cell differentiation [132].

KGF (keratinocyte growth factor), also known as FGF7, is a member of the fibroblast growth factor family that can stimulate ductal cell proliferation [146]. It has also been observed that in rats, KGF acts on ductal cells by activation of distinct signaling pathways to promote *β*-cell regeneration [141]. KGF is widely used in stepwise differentiation media as it can generate both PDX1 + and subsequent PDX1 + /NKX6.1 + pancreatic progenitors populations, respectively [75, 147–149]. Activation of protein kinase C (PKC) is reported to induce pancreatic precursors during *β*-cell differentiation protocols [75], PKC activation increases *β*-cell proliferation, size and mass *in vivo* and is required for growth factor-stimulated *β*-cell proliferation in vitro [150, 151].

### Pancreatic endocrine cells

After induction of pancreatic precursors cells, the in vitro differentiation process must be centered on obtaining endocrine cell specification. Endocrine differentiation is initiated in PDX1 + /NKX6.1 + progenitor through inhibition of Notch signaling, allowing the expression of *ngn3*, as previously described [74, 152]. These authors reported that cells undergoing endocrine differentiation lose responsiveness to Notch, because Notch activation in Ngn3 + endocrine precursors prevents their differentiation. Ngn3 + cells are promising candidates for endocrine progenitor cells, since they display proliferative capacity and generate cells that express islets-specific transcription factors, such as NEUROD, NKX6.1 and PAX6 [110]. At this stage, using distinct combinations of transcription factors, a specific gene expression profile is initiated and maintained, allowing specification of multipotent progenitors toward the differentiated lineages [112]. Initially, D’Amour et al. [74] included DAPT (gamma secretase inhibitor), a Notch pathway inhibitor, to obtain NGN3 + cells, but it was later shown that it may have a slight beneficial effect on this differentiation step. Despite that, other studies demonstrated that DAPT could have an important role in inducing islet-like structures from embryonic pancreatic precursor cells [153] and several differentiation protocols included the Notch inhibitor *γ*-secretase inhibitor II [75, 154, 155]. Retinoic acid is proposed to expand the endocrine cell population and block the formation of exocrine cells in a dose-dependent manner [134, 137, 156]. Also, it has been shown that activin A enhances transcription of the *ngn3* gene through Smad4 (TGF-*β*/Smad pathway), binding to the promoter region of *ngn3* [157].

It is well known that thyroid hormones have several effects in the development of many endocrine glands, including pancreas [158]. Aiello et al. [158] showed that the thyroid receptors TR*α*1 and TR*β*1 mRNAs were differentially expressed at different phases of embryonic murine pancreas development. These authors found increased mRNA levels of the pro-endocrine gene ngn3 (and increased number of *β*-cells in cultures previously treated with Triiodothyronine (T3)). The mechanism of T3 action was found to be induction of acinar reprogramming into ductal-like cells that subsequently will differentiated into endocrine cells [112]. Some studies indicate that the Pdx1 + progenitor cells differentiation process requires two major events after establishment of the definitive endoderm, in order to mimic the in vivo process, namely blocking liver differentiation induction by BMP antagonism and induction of pancreatic progenitors by the retinoic signaling pathway [159]. The alternative hepatic lineage differentiation path can be inhibited by treating the cells with different types of inhibitors, such as NOGGIN (BMP antagonist) [152, 160]. It has been demonstrated that the combination of EGF and nicotinamide signaling, together with inhibition of the BMP pathways, promotes an efficient development of NKX6.1 + progenitors from hiPSC lines [143]. The BMP antagonism requirement should be reversed after induction of the pancreatic cell lineages, since BMP signaling is necessary for maintenance of *pdx*1 expression and additional cell differentiation later on, which can be complicated to implement during in vitro differentiation [161, 162].

Nostro et al. and Chen et al. [135, 163] showed that the inhibition of the TGF*β*/activin/nodal and BMP pathways by adding the small molecule ALK4/5/7 inhibitor SB431542 (SB) and Noggin immediately following PDX1 induction had an additive effect, resulting in a sixfold increase in INS expression over that observed in untreated cultures. The results indicated that inhibition of TGF*β*/activin/nodal and BMP signaling following induction of pancreatic progenitors does promote differentiation to the endocrine lineage. Rezania et al. [154] and Pagliuca et al. [75] reported that ALK5i and T3 played a significant role at late stages of the differentiation protocol to generate stem cell-derived *β*-cells. However, Velazco-Cruz et al. [164] identified that inhibiting TGF-*β* signaling during the last stage of the protocol greatly reduces the function of these differentiated cells, while treatment with Alk5i during the previous stage is necessary for a robust *β*-like cell phenotype.

### Mature *β*-cells

The final stage of *β*-pancreatic differentiation aims at *β*-cells specification and maturation to obtain high levels of cells displaying glucose-stimulated insulin secretion capacity. Mature adult pancreatic *β*-cells are functionally defined by their rapid response to elevated glucose [154]. To reach this cellular profile, frequently, factors and molecules that are known to act in adult pancreas are used. Betacellulin, a member of the epidermal growth factor family, is produced by proliferating pancreatic *β* cells [135] and can increase Pdx1 and insulin production [165]. At this stage, nicotinamide supplementation is usually added to the culture medium, to enhance the in vitro differentiation of cultured human pancreatic cells, favoring the expression of insulin, glucagon and somatostatin [166]. Nicotinamide has historically been used to augment pancreatic *β*-cell differentiation and to protect islet cells from toxic insults, due to its antioxidant properties. Studies showed that cells exposure to nicotinamide is essential for robust *nkx6.1* expression in hiPSC differentiation to pancreatic endocrine progenitors, acting predominantly through PARP (poly-ADP-ribose polymerases) inhibition [167, 168]. Thowfeequ et al. [169] showed that the addition of betacellulin and nicotinamide to the modified differentiation protocol sustained PDX1 expression and induced pancreatic *β*-cell differentiation in human ES cell line.

Glucagon-like peptide-1 (GLP-1) is an intestinal incretin hormone that binds to specific G protein-coupled receptors on pancreatic *β*-cells to stimulate insulin secretion via cAMP-dependent pathways. Consequently, GLP-1 plays a crucial role in *β*-cell mass regeneration [170]. Exogenous GLP-1 increases islet cell proliferation in Ins-1 cells via a PI3-kinase-dependent pathway [171]. Exendin 4, a long-acting GLP-1 analogue, is resistant to dipeptidyl peptidase IV (DPP-IV) cleavage, being more useful clinically, and can also be used to promote *β*-cell proliferation. Considering the importance of *mafA* expression in *β*-cells, representing an important indicator of *β*-cell maturity, studies identified that thyroid hormone is also a physiological regulator of *β*-cell maturation through direct interaction with the *mafA* promoter [172]. Therefore, thyroid hormone may improve in vitro functional maturation of immature stem cells-derived insulin-expressing cells. Moreover, it is believed that VEGF (vascular endothelial growth factor) is predominantly secreted by *β*-cells in adult pancreas, affecting islet function and physiology [173]. Consequently, exogenous supplementation with VEGF has been associated with reduction in *β*-cell apoptosis and maintenance of *β*-cell mass [174].

Another important component that is crucial and should be provided during *β*-cell differentiation and maturation is the major components of the extracellular matrix (ECM) of islet cells, including laminin and collagen. The islet ECM has been shown to regulate survival, insulin secretion, proliferation and islet morphology. Moreover, laminin and type IV collagen were identified to be beneficial for *β*-cell function in vitro [175]. Laminins were shown to induce expression of islet-specific transcription factors and hormones, such as Pdx1, insulin1, insulin2, glucagon and Glut2 [176]. In in vitro experiments, collagen has been associated with provide the desired mechanical properties of transplanted grafts, to improve the performance of scaffolds and, in combination with other ECM proteins, such as laminin, to enhance glucose-stimulated insulin secretion in pancreatic islets [177, 178]. Therefore, providing islet matrix proteins to the in vitro differentiation process is a key determinant for presentation of matrix-bound signals, warranting a microenvironment which is closer to the native in vivo situation, thereby sustaining the maintenance of cellular viability.

## Challenges and achievements

Although several factors are important for successful generation of IPCs from iPSCs or ESCs, careful handling of cell culture conditions stands out as one of the most critical factors [161]. Table 3 highlights the main growth and differentiation factors used during the four critical steps of *β*-cell differentiation from hiPSC or hESC, described in major reports found in the literature. Numerous efforts have been employed to obtain hPSC-derived *β*-cells since Lumelsky et al. [72] first described a protocol to enrich IPC from ESCs by selecting NESTIN + cells, but only in 2014 two different research groups [75, 154] published a protocol showing the differentiation of human embryonic stem cells (hESCs) into *β*-cells that resemble cadaveric *β*-cells with respect to both gene expression and function. It was quite a breakthrough in developing stem cell-derived *β*-cells, and currently, the protocol developed by the Melton Lab [75] is the basis for the Vertex clinical trial therapy.

**Table 3:** Differentiation factors from major reports in the literature regarding stem cell-derived *β*-cell

Reference	Cell type	Definitive Endoderm	Pancreatic precursor	Endocrine precursor	IPCs/Endocrine cell	Outcomes
[74]	CyT203 hESC	Activin A + WNT3a	FGF10 + CYC + RA (d9)	DAPT + Ex4	Ex4 + IGF1 + HGF	12% of the cells insulin + ; Polyhormonal cells; Glucose-induced C-peptide release consistent with adult islets
[179]	H1, H7, and H9 hESCs	Activin A + NA-Butyrate	EGF + bFGF + Noggin	EGF + bFGF (14d) + Noggin	Nicotinamide + IGF-II	2%–8% human C-peptide containing cells; Polyhormonal ILCs released C-peptide in a glucose-dependent manner
[180]	H1 and H9 hESCs	Activin A + CDM	RA + CDM (4d) + bFGF		Nicotinamide + bFGF	> 15% of cells were C-peptide + C-peptide content increased by 100% with high glucose 30% of the transplanted mice exhibited stable euglycemia for more than 6 weeks
[137]	CyT203 hESCs	Activin A + Wnt3a (1d)	FGF10 + RA	No factors	Transplantation in immunodeficient mice	Polyhormonal (> 50% endocrine cells) C-peptide release for > 150 days after engraftment; 92%of implanted mice achieved protection against STZ-induced hyperglycemia
[76]	H9 and H1 hESCs and hiPSC	Activin A + Wortmannin	RA + FGF7 + Noggin	EGF	Nicotinamide + bFGF + Exendin 4 + BMP4	Approximately 25% of insulin + cells; Cells secreted C-peptide in response to KCl stimulation C-peptide release increased by high glucose comparable to adult human islet
[141]	253G1 hiPS cell line	Activin A + CHIR (1d) + Wnt3a (1d)	Noggin + Dorsomorphin RA + SB431542		Forskolin + Dexamethasone + Alk5i + Nicotinamide	7.8% were C-peptide + /glucago- cells Secretes C-peptide in response to various stimuli, but had no GSIS
[75]	HUES8 hESCs, hiPSC-1 and hiPSC-2	Activin A + CHIR	KGF + RA + SANT1 + LDN + PdbU	RA + SANT1 + Heparin + Betacellulin + XXI + T3 + Alk5i	T3 + Alk5i	75% of SC-*β* cell clusters responded to high glucose challenges; 33% of NKX6-1 + /C-peptide + cells; Mice transplanted maintained insulin secretion for 18 weeks
[181]	KhES-3 (hESC) 253G1 (hiPSC)	Activin A + CHIR + Wnt3a (2d)	FGF10 (7d) + Noggin + Dorsomorphin + RA + FR180204		Forskolin + Dexamethasone + Alk5i + nicotinamide	30% of insulin-producing cells INS + cells secreted insulin in response to glucose
[154]	H1 hESC and hiPSC	GDF8 + GSK3*β* inh	FGF7 + Ascorbic acid + RA + SANT + TPB + LDN	SANT + RA + Alk5i + T3 + LDN	Alk5i + T3 + LDN + XXI + N-Cys + AXLi	50% of cells were insulin + with the vast majority PDX1 + and NKX6.1 + At 16 days post-transplantation BG were reduced in mice; BG were maintained by 60 days post-transplant
[182]	CyT49 hESC	Activin A + Wnt3a (1d)	KGF + EGF Noggin + Activin A + Heregulin-*β*1 + Wnt3a + TTNPB + CYC	ROCKi + KGF + EGF + GSI + Nicotinamide	Nicotinamide + Matrigel + ROCKi + T3	73–89% endocrine cells, 40%–50% expressed insulin Increased GSIS at 12 weeks after transplantation
[135]	H1 and H9 hESC	Activin A + FGF2 + CHIR/Wnt3a	FGF10 + Noggin + CYC + RA	Noggin + EGF + Nicotinamide	–	83.1 ± 4.2 of efficiency of differentiation for H1 cell line; High levels of C-peptide after glucose challenge in transplanted mice at 18 weeks
[152]	MEL1 INSGFP/W hESC	ITS + Activin A + WNT3a	CYC + RA + EGF + KGF	TBP + ALKi + Noggin + KGF	–	23% of *β*-like cells in differentiated cells; Reduction in mice BG levels; Lack of complete DM reversal in mice;
[183]	H1 and CHA15 hESCs and hiPSC line	Activin A + CHIR (1d) + LiCl (1d)	RA + Dorsomorphin + SB431542 bFGF + KAAD CYC	DAPT + Dorsomorphin + SB431542 + Ascorbic acid	Dibutyryl-cAMP + Exendin 4 + SB431542 + Dorsomorphin + Nicotinamide + Ascorbic acid	Increased insulin secretion with high glucose Decrease in mice BG level within 3 days post-transplantation Regulation of BG in mice for 12 days
[184]	SR1423 Cell line^a^	Activin A + Wortmannin	RA + KGF + Noggin + CYC	Noggin + EGF (10d) + KGF (10d) + XXI + Alk5i + RA (16-18d)	Alk5i + Nicotinamide + IGF-I + BMP4 + T3	60% insulin-expressing cells that secrete insulin in response to glucose Lower BG in transplanted mice within 7 ± 21 days and was maintained for weeks
[164]	HUES8 cell line	Activin A + CHIR	KGF + RA + SANT1 + ROCKi + LDN + PdbU (1d) + Activin A (8-12d)	RA + SANT1 + T3 + XXI + Alk5i + Betacellulin	ESFM + Cluster resize	96% of cells expressed CHGA and 73% expressed C-peptide; Maintenance glucose tolerance after 10 weeks of mice transplantation
[185]	ChiPSC12 and ChiPSC22; HuES8; H1ES; hiPSCs from HUVEC	Activin A + CHIR (1d)	Ascorbic acid + FGF7 + SANT1 + RA + LDN + ALK5i +	Ascorbic acid + XXI + T3 + ALK5i + LDN	Ascorbic acid + Trolox + AXLi + XXI + Alk5i + T3 + WNT4 + Laminin	50–60% of cells co-expressed insulin and *β*-cell markers Glucose homeostasis in mice for 50 days Overexpression of PD-L1

*Alk5i* Alk5 receptor inhibitor II, *AXLi* ALX inhibitor, *Betacellulin* EGF family member, *BG* Blood glucose, *CDM* chemically defined medium, *CHGA* chromogranin A, a pan-endocrine marker, *CHIR* GSK3a/b inhibitor (can substitute Wnt3a), *CYC* Cyclopamine, *ECC* Endocrine cells clusters, *FR180204* inhibit the kinase activity of ERK1/2 as well as TGF-*β* induced AP-1 activation, *ESFM* enriched serum-free medium, *GDF8* a TGF*β* family member, *IGF-II* insulin-like growth factor, *ILC* islet-like cells, *KGF* keratinocyte growth factor LDN LDN193189, a BMP type 1 receptor inhibitor, *LiCl* Lithium Chloride—can affect cell signaling pathways such as WNT-Frizzled, *N-cys* *N*-acetyl cysteine, *PdbU* Phorbol 12,13-dibutyrate, a protein kinase C activator, *PD-L1* programmed death-ligand 1, *RA* retinoic acid SANT1 Hedgehog inhibitor, *SC-β* stem-cell-derived b cells, *T3* triiodothyronine, a thyroid hormone, *TBP* TATA box binding protein, *TGFβi* TGF*β* RI Kinase inhibitor IV, *Trolox* derivative of vitamin E, *TTNBP* retinoic acid analog, *XXI* *γ*-secretase inhibitor, *α-APPM* *α*-amyloid precursor protein modulator

^a^iPSC from islets primary cells screened for endodermal markers and pancreatic progenitor markers—the cell line that consistently generated the highest proportion of pancreatic cells was named SR1423

Acquisition of dynamic insulin secretion upon glucose stimulation is a key feature of *β*-cells. This dynamic function is represented by a pulsatile behavior of two-phase insulin secretion: The first phase has a period of 10–15 min following stimulation by glucose, comprising a high amplitude but with short duration, while the second phase has a lower amplitude and a longer duration of 1–2 h [186, 187]. According to Table 3, many of the protocols generated immature mixed populations of cells at different developmental stages, displaying polyhormonal properties and, additionally, IPCs-transplanted mice usually maintain euglycemia for only a short period of time or present a compromised GSIS dynamics. Indeed, the majority of *β*-like cells derived from stem cells differentiation resemble fetal *β*-cells regarding their maturity [188]. Velasco-Cruz et al. [164] first reported robust dynamic insulin secretion of SC-*β* cells. It was further shown that manipulation of the polymerization state of actin cytoskeleton influences NEUROG3-dependent endocrine induction. The results obtained allowed overcoming the requirement for three-dimensional culture in stem cell-derived *β*-cell differentiation and creating a fully planar protocol [189]. These findings enable simplifying the differentiation methodology, requiring only basic stem cell culture experience, as well as familiarity with assessment techniques which are commonly used in biology laboratories [190].

Nair et al. [191] optimized the Russ et al*.* protocol [152] to increase *β*-like cells maturity through reaggregation of INS^+^ *β*-like cells isolated by fluorescence-activated cell sorting (FACS); however, these cells presented a marked first phase response to glucose but failed to sustain the second phase of insulin secretion. Studies by Yoshihara et al. [192] demonstrated that stem cells-derived *β*-cells could acquire adult insulin secretion behavior through overexpression of estrogen-related receptor *γ* (ERR*γ*), which is hypothesized to regulate mitochondrial metabolic pathways required for GSIS. In the attempt to characterize the protocol of in vitro differentiation, single-cell transcriptome has been undertaken to visualize populations and pathways regulated during the stages [193].

It is important to highlight that native pancreatic islet is highly vascularized cellular aggregates, consisting of, approximately, 10% of blood vessels, which are essential to allow networking between glucose concentration sensing and insulin secretion by *β*-cells and, also, to provide proper islet oxygenation [194]. The lack of these vasculature interactions is one of the main reasons for the low survival rate of transplanted islets [195]. In this context, studies have hypothesized that in vitro interaction between ESC-derived EBs and endothelial cells may augment the differentiation toward pancreatic endocrine progenitors and IPCs [196]. Weizman et al*.* [197] also proposed a 3D architecture system using polymeric scaffolds to culture hESC-derived pancreatic cells embedded in a vascular niche composed of endothelial cells and/or fibroblasts. Therefore, endothelial cells may provide key factors that lead to the endocrine cell fate during in vitro differentiation. In general, incorporation of endothelial cells and other important cells normally present in the *β*-pancreatic niche may be beneficial for improving IPCs differentiation and functionality.

Pancreatic islets also receive complex neural inputs, and *β*-cells present a phenotypically diverse population, with a mosaic of metabolic and electrical activity patterns [198]. Although adult *β*-cells populations are totally differentiated, they are heterogeneous with respect to their insulin secretory abilities, mitochondrial function, calcium signaling and proliferative properties. For this reason, maturity is not defined only by the expression of major molecular markers, such as PDX1, NKX6.1 and MAFA, or by high insulin expression levels [41]. Johnston and colleagues [199] have reported that a 5–8% subset of *β*-cells forms “super-connected hubs” within an interconnected islet cellular network. It has also been shown that these cells serve as pacemakers that can synchronize the calcium and insulin secretory responses across the whole islet. In addition, *β*-cells can be divided into two major populations: One comprised of cells that are capable of proliferation and the other one comprised of mature *β*-cells that are marked by the expression of Fltp (also known as Flattop or Cfap126), a Wnt/PCP (planar cell polarity) effector. FLTP + cells represent the subpopulation of mature *β*-cells, while Fltp-negative cells comprise immature and proliferative cells [200]. However, Dorrell et al*.* [200] demonstrated that human *β*-cells have at least four different cellular subtypes, which may be classified based on their cell surface markers expression. This suggests a functional heterogeneity among *β*-cells and illustrates the degree of complexity of the insulin release kinetics that stem cells-derived IPCs should probably achieve.

Typically, a patient requires two transplants, each of which with at least 10.000 islet “equivalents” (IEQs) per kilogram of body weight, to achieve insulin independence [19]. Proportionately, a single 70 kg patient requires approximately 700 million of transplanted IEQs [19]. This poses important challenges related to manufacturing sufficiently pure and potent cells, at scale, for clinical use and, also, protecting these cells from immune rejection following transplantation. Some strategies to address these limitations have already been described. Schulz et al*.* [149] reported a process that allows scaled production of hESC and, subsequently, of pancreatic progenitors. These authors developed a feeder-free culture system for expansion of the CyT49 hESC line and generation of large-scale single-cell master and working banks of CyT49 under good manufacturing practices (cGMP) [149].

Although autologous transplantation of patient-specific IPCs derived from iPSCs emerged as an attractive strategy, it still requires suppression of the preexisting autoimmunity [201, 202]. The negative effects of some immunosuppressants in human *β*-cell transplantation patients have been widely reported, being associated with complications at new onset DM upon transplantation [203–205]. Another interesting approach is to mediate genetic manipulation in order to control the expression of HLA class I and II genes, allowing the graft to escape from immune recognition and destruction [206]. Furthermore, Yoshihara et al*.* [185] showed that human islet-like organoids (HILOs) generated from iPSCs overexpressing PD-L1, a known determinant of immune tolerance in *β*-cells, are protected from xenograft and allogenic rejection and maintain glucose homeostasis in diabetic mice.

The encapsulation strategies are currently the most promising approach, representing the most adequate alternative, when compared to adoption of the immunosuppressive regimen. Cell encapsulation creates a physical barrier for the transplanted IPCs, providing a 3D architecture that may attenuate the deleterious impact of the host immune system on newly transplanted cells [207]. Encapsulation of pancreatic islets with artificial membranes allows preservation of their physical characteristics and functional integrity. Furthermore, studies carried out by our group demonstrated that incorporation of polylaminin into the microcapsule polymer attenuated the post-transplantation immunological response against microcapsules grafted in mice, suggesting an improved maintenance of the grafted encapsulated pancreatic islets in the recipient organism [14]. Vegas et al*.* [208] carried out an experiment of long-term evaluation of encapsulated SC-derived *β* cells in immune-competent mice. They showed that stem cell-derived *β*-cells can promote long-term glycemic correction (174 days) in an immune-competent diabetic animal in the absence of immunosuppressive therapy, using a modified alginate capable of mitigating the innate immune-mediated foreign body responses, with euglycemic mice still being present at the end of the experiment. Subsequently, the same chemically modified alginate, called Z1-Y15, was shown to prevent pericapsular fibrotic overgrowth and maintain encapsulated islets function after four months of allogenic transplantation in non-human primates, in the absence of immunosuppression, in a pre-clinical study [209]. These authors also suggest an alternative transplantation site into the bursa omentalis, which can support nutritional exchange for long-term islet viability. This technology was incorporated by the Sigilon Therapeutics company and has already been tested in clinical trials (phase 1/2) to assess the safety, tolerability and preliminary efficacy of SIG-001, which is composed of human cells that are engineered to produce FVIII, in adults with severe or moderately severe hemophilia A [210].

An important concern with stem cells-derived therapeutic products is the presence of undifferentiated or partially differentiated cells that may not only interfere with the desired cell types activity, but, also, be tumorigenic. For this reason, optimization of the in vitro differentiation process is fundamental to minimize the formation of unwanted cell types and, consequently, validate this technology for clinical use [44]. Additional approaches to eliminate non-differentiated cells include the use of antibody-toxin molecules or conjugates that selectively kill non-differentiated cells [211]. Despite the existing risks, many different strategies have been employed to promote in vivo maturation of transplanted progenitor cells [137, 212]. However, the use of encapsulation devices that provide their precise location in the body and the possibility to be recovered in case of graft failure or other complications is a promising approach to allow safe progenitor cells transplantation [182, 213]. In general, the choice of cells at different stages of maturation has many safety-related implications, with mature differentiated cells being the safest ones since they display low levels of residual plasticity [214].

Many advances have been made with respect to the establishment of differentiation protocols capable of generating homogeneous cell masses at early stages of development. Also, many efforts have been made to generate better functioning *β*-cells by introducing some features that could favor the differentiation process, such as promoting clustering of immature *β*-like cells into endocrine-enriched niches [191], assembly of islet-like organoids onto hydrogel slabs [82, 215], engineering human islet organoids using an organ-on-a-chip platform [216] and culturing in decellularized pancreatic scaffolds [217]. However, a standardized differentiation protocol is still lacking, and the final differentiation stages also need to be better understood. To address this challenge, understanding the whole transcriptome, epigenome and proteome of the differentiation process could help to obtain insights into the pathways that lead to the process of mature and functional *β*-cells generation.

## Conclusions

In conclusion, generation of pancreatic *β*-cells from pluripotent stem cells constitutes a very promising therapeutic approach to provide insulin independence to millions of diabetic patients. Differentiation protocols, cell culture methodology and encapsulation protocols are being developed to optimize *β*-cells production and provide protection against the autoimmune response displayed by T1D patients. Although several previously mentioned challenges still need to be overcome, a great deal of efforts has been employed combining several interdisciplinary fields, such as stem cell biology, embryology, immunology, cell encapsulation and tissue bioengineering, to enable the development of effective cellular therapies.

## Data Availability

Not applicable.

## Abbreviations

ANVISA: Brazilian Health Regulatory Agency
ASCs: Adult stem cells
ATG: Anti-thymocyte globulin
BPRs: Batch production records
cGMP: Current good manufacturing practices
DM: Diabetes mellitus
EBs: Embryoid bodies
ECM: Extracellular matrix
EMA: European medicines agency
ESCs: Embryonic stem cells
FACS: Fluorescence-activated cell sorting
FDA: Food and drug administration
GAD65: Glutamic acid decarboxylase 65
HILOs: Human islet-like organoids
IA-2: Islet antigen 2
IDF: International diabetes federation
IEQs: Islet “equivalents”
IPCs: Insulin-producing cells
iPSCs: Induced pluripotent stem cells
MBRs: Master batch records
MSCs: Mesenchymal stem cells
MTT: Meal tolerance test
OGT: Oral glucose tolerance test
pMHCII: MHC-II/peptide complex molecules
SCs: Stem cells
SOP: Standard operating procedures
T1D: Type 1 diabetes mellitus
T2D: Type 2 diabetes
Teffs: Effector T cells
Tregs: Activated regulatory T cells
WHO: World Health Organization
ZnT8: Zinc transporter 8
