# Correction: Stem cells differentiation into insulin-producing cells (IPCs): recent advances and current challenges

**DOI:** 10.1186/s13287-022-03206-2

**Published:** 2022-11-15

**Authors:** Isaura Beatriz Borges Silva, Camila Harumi Kimura, Vitor Prado Colantoni, Mari Cleide Sogayar

**Affiliations:** 1 Cell and Molecular Therapy Center (NUCEL), School of Medicine, University of São Paulo, São Paulo, SP 05360-130 Brazil; 2 Department of Biochemistry, Chemistry Institute, University of São Paulo, São Paulo, SP 05508-000 Brazil

## Correction to: Stem Cell Research & Therapy (2022) 13:309 https://doi.org/10.1186/s13287-022-02977-y

On page 8 of the original article [1], in the sentence: "… differentiation of iPSCs in pancreatic β-cells, namely Wnt; Nodal/Activin A; BMPs; FGF; EGF (epidermal growth factor); Hedgehog; retinoid; and Notch (Fig. 1).". This figure indication is incorrect; the correct figure corresponding to this sentence is Fig. 2.
